# A high solids field-to-fuel research pipeline to identify interactions between feedstocks and biofuel production

**DOI:** 10.1186/s13068-021-02033-6

**Published:** 2021-09-10

**Authors:** Meenaa Chandrasekar, Leela Joshi, Karleigh Krieg, Sarvada Chipkar, Emily Burke, Derek J. Debrauske, Kurt D. Thelen, Trey K. Sato, Rebecca G. Ong

**Affiliations:** 1 DOE Great Lakes Bioenergy Research Center, Michigan Technological University, Houghton, MI USA; 2 Department of Chemical Engineering, Michigan Technological University, Houghton, MI USA; 3 DOE Great Lakes Bioenergy Research Center, Univ. of Wisconsin-Madison, Madison, USA; 4 DOE Great Lakes Bioenergy Research Center, Michigan State University, East Lansing, MI USA

**Keywords:** Enzymatic hydrolysis, High solids loading, Horizontal tumbling, Fermentation, Field-to-fuel, Environmental factors, Abiotic stressors

## Abstract

**Background:**

Environmental factors, such as weather extremes, have the potential to cause adverse effects on plant biomass quality and quantity. Beyond adversely affecting feedstock yield and composition, which have been extensively studied, environmental factors can have detrimental effects on saccharification and fermentation processes in biofuel production. Only a few studies have evaluated the effect of these factors on biomass deconstruction into biofuel and resulting fuel yields. This field-to-fuel evaluation of various feedstocks requires rigorous coordination of pretreatment, enzymatic hydrolysis, and fermentation experiments. A large number of biomass samples, often in limited quantity, are needed to thoroughly understand the effect of environmental conditions on biofuel production. This requires greater processing and analytical throughput of industrially relevant, high solids loading hydrolysates for fermentation, and led to the need for a laboratory-scale high solids experimentation platform.

**Results:**

A field-to-fuel platform was developed to provide sufficient volumes of high solids loading enzymatic hydrolysate for fermentation. AFEX pretreatment was conducted in custom pretreatment reactors, followed by high solids enzymatic hydrolysis. To accommodate enzymatic hydrolysis of multiple samples, roller bottles were used to overcome the bottlenecks of mixing and reduced sugar yields at high solids loading, while allowing greater sample throughput than possible in bioreactors. The roller bottle method provided 42–47% greater liquefaction compared to the batch shake flask method for the same solids loading. In fermentation experiments, hydrolysates from roller bottles were fermented more rapidly, with greater xylose consumption, but lower final ethanol yields and CO_2_ production than hydrolysates generated with shake flasks. The entire platform was tested and was able to replicate patterns of fermentation inhibition previously observed for experiments conducted in larger-scale reactors and bioreactors, showing divergent fermentation patterns for drought and normal year switchgrass hydrolysates.

**Conclusion:**

A pipeline of small-scale AFEX pretreatment and roller bottle enzymatic hydrolysis was able to provide adequate quantities of hydrolysate for respirometer fermentation experiments and was able to overcome hydrolysis bottlenecks at high solids loading by obtaining greater liquefaction compared to batch shake flask hydrolysis. Thus, the roller bottle method can be effectively utilized to compare divergent feedstocks and diverse process conditions.

**Supplementary Information:**

The online version contains supplementary material available at 10.1186/s13068-021-02033-6.

## Background

Liquid transportation fuels from lignocellulosic biomass can play a vital role in reducing greenhouse gas emissions and mitigating climate change [1]. Environmental factors experienced during plant growth, such as weather extremes, have the potential to cause adverse effects on biomass quality and quantity [2, 3]. Decreased yield due to drought is a serious challenge to uninterrupted supply of feedstock for biofuel production [4–6]. Several studies have focused on the effects of abiotic stressors, such as drought, extreme temperatures, and heavy metal and salt concentrations on feedstock yield and composition to identify ecosystems suitable for their cultivation and develop sustainable bioconversion processes [7–9]. Although environmental factors can also affect the deconstruction of feedstocks into biofuel and resulting fuel yields, only a few studies have evaluated these effects [10–12]. There are a number of challenges in conducting these field-to-fuel experiments that span the entire biofuel production chain. First, a thorough analysis of the environmental effect on feedstocks and subsequent correlation to biofuel production requires samples from multiple plots and locations, which needs a higher level of throughput for the process than can be achieved in bioreactors. Second, there is a limit on minimum scale for reliable (useful or interpretable) fermentation experiments in order to be comparable to experiments performed in bioreactors. This requires a minimum hydrolysate volume and moderately sized pretreatment and hydrolysis vessels. Thus, there is a need for a platform that is able to accommodate a larger number of samples, while generating sufficient volumes of hydrolysate in a reasonable time frame.

Laboratory-scale enzymatic hydrolysis for screening numerous lignocellulosic materials is usually performed at a substrate solid loading of 1 wt% in a vial or up to 5 wt% in a shake flask [13]. However, high solids loading hydrolysis (18 wt% or higher) is needed to more accurately represent industrial conditions. Enzymatic hydrolysis at high solids loading increases the economic feasibility of the bioconversion process as it reduces the operating cost for hydrolysis and fermentation and minimizes energy requirements for other downstream processes, such as distillation [11, 14, 15]. However, as solids loading increases, the water available to facilitate the diffusion of enzymes into the biomass and the diffusion of sugars out into solution decreases [16–18]. Water availability is also important to reduce the viscosity of the slurry, thereby reducing the energy required for mixing. Poor mixing due to low water availability is a significant bottleneck for high solids loading operating conditions [19–21]. Shake flasks are commonly used lab equipment for enzymatic depolymerization of biomass, but they do not provide sufficient shear rates to reduce viscosity at high solids concentrations. Inadequate mixing results in hydrolysis product build up in specific areas of the flask and improper enzyme distribution. In a shake flask, the highly viscous biomass and water mixture accumulates near the walls of the flask, which is the low shear zone of the reaction vessel [22, 23]. In contrast to orbital shaking, gravitational tumbling has been found to be effective in mixing under high solids conditions [24–26]. Fed-batch, high solids loading enzyme hydrolysis has also been studied extensively as a means to overcome mixing issues [27–29]. In this method, the experiment is started with a moderate amount of the biomass, and a small dose of biomass is added at regular intervals. Fed batch optimizes the inherent pseudoplastic behavior of the high solids slurry by improving the water availability for enzymatic hydrolysis [30, 31]. However, fed-batch loading also increases the likelihood of contamination if not conducted in a controlled manner.

The objective of this project was to develop a laboratory-scale high solids field-to-fuel platform to evaluate fermentation performance of diverse feedstocks. Custom pretreatment reactors were designed to process sufficient AFEX treated biomass to generate the volume of high solids hydrolysate required for fermentation [32]. Enzymatic hydrolysis was conducted using gravitational tumbling in a static incubator to ensure proper mixing under high solids loading conditions and compared to the conventional method using shake flasks. Enzymatic hydrolysis parameters, including solids loading, buffer concentration, and pH, were optimized to achieve the highest volumes of hydrolysate and sugar conversion. The hydrolysates were fermented using *Saccharomyces cerevisiae* or *Zymomonas mobilis* with a system that measures real-time CO_2_ production in order to determine fermentation rate without extensive manual sampling and culture depletion. The fermentation performance of the platform was validated using two switchgrass samples that previously have shown divergent fermentation performance when processed at a larger scale for all steps––pretreatment, hydrolysis, and fermentation. This field-to-fuel platform can be used to rapidly identify the effects of environmental conditions, genetic background, or other parameters that influence feedstock quality, on microbial fuel production under industrially relevant enzymatic hydrolysis and fermentation conditions.

## Results

### Feedstocks and pretreatment

Correlating environmental field conditions to effects on feedstock deconstruction and fuel production will require analysis of a large number of samples that control for multiple variables (e.g., local temperature and precipitation, soil type, and field location). This study used a variety of potential herbaceous bioenergy feedstocks (corn stover, switchgrass, sorghum, restored prairie, and miscanthus), to demonstrate the broad utility of the platform. The majority of feedstocks in this study were pretreated in a larger Parr reactor, in order to have a consistent supply of feedstock for developing the enzymatic hydrolysis method. However, the actual pipeline makes use of smaller custom AFEX reactors that can pretreat 25 g of lignocellulosic biomass per batch [32]. Once the hydrolysis method was finalized, the custom reactors were used to process two feedstocks with previously observed divergent fermentations and validate the method.

### Enzymatic hydrolysis buffer pH and concentration influence sugar release and fermentability of the hydrolysates

Common hydrolysis bottlenecks in shake flasks at high solids loading include insufficient shear rate, improper enzyme distribution, and inadequate mixing due to accumulation of biomass near the walls. A laboratory-scale roller bottle hydrolysis method was developed in order to overcome these bottlenecks. Hydrolysate pH significantly affects liquefaction and sugar yields due to its influence on enzyme activity. The enzymes used in our study were from Novozymes Ctec and Htec series, which have cellulase, hemicellulase, xylanase, and betaglucosidase activities [33–35]. The activities of our cellulolytic enzymes have an optimal pH range of 5.0 to 5.5 [36]. However, AFEX pretreated biomass when stored in liquid water (either before or after autoclaving) has a pH of ~ 7, which means a pH adjustment step is necessary for effective enzymatic activity. We initially tested pH control by adding HCl following autoclaving. However, because of the variability between feedstocks, it was difficult to estimate the amount of HCl required to reach the desired pH, which meant that pH adjustment became a long and laborious process. For a platform that was intended to work on hundreds of feedstocks with unknown native buffering capacity, it was decided that this approach was impractical. Instead, we decided to adjust pH using phosphate buffer, which was chosen based on its use in a previous study on fermentation of AFEX hydrolysates [37].

**Fig. 1:**
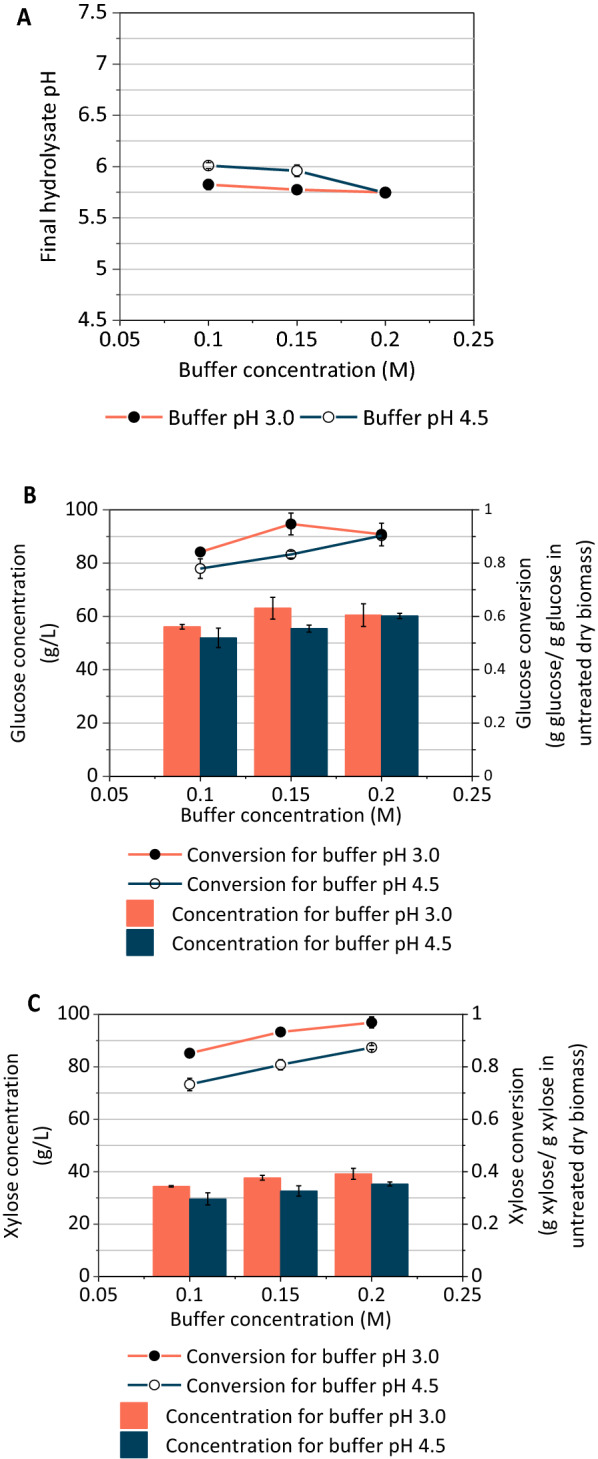
**A** Increasing buffer pH and concentration decreased hydrolysate final pH. **B** Increasing buffer pH (3.0 to 4.5) and concentration (0.1 to 0.2 M) increased glucose conversion and glucose concentration for AFEX pretreated CS at 6% glucan loading. **C** Increasing buffer pH (3.0 to 4.5) and concentration (0.1 to 0.2 M) increased xylose conversion and xylose concentration for AFEX pretreated CS at 6% glucan loading for buffer pH 3.0 and 4.5. Values for all subfigures are reported as mean ± SD, *n* = 2

The effect of buffer pH and concentration on hydrolysate characteristics was evaluated using 6% glucan loading (19% w/w solids loading) AFEX corn stover hydrolysates. The final hydrolysate pH was lower for pH 3.0 buffer compared to 4.5, and for all concentrations except 0.2 M (Fig. 1A). While the pH 4.5 buffer showed an effect of concentration on final hydrolysate pH, this was not observed for the pH 3.0 buffer, which had a consistent final pH of ~ 5.75 regardless of buffer concentration. All hydrolysates had a pH ranging between 5.75 and 6.0, which was slightly higher than optimal enzyme activity, but in a suitable range for *Z. mobilis* fermentations, which meant that less pH adjustment was required following enzymatic hydrolysis. Although the hydrolysate pH was not strongly affected by buffer concentration and pH, both of these properties affected carbohydrate conversion and the fermentability of the hydrolysates (Fig. 1), with the glucose conversion consistently higher for a buffer pH of 3.0 than 4.5. The highest glucose conversion was attained for the buffer concentration of 0.15 M and pH of 3.0 (Fig. 1A).

**Fig. 2:**
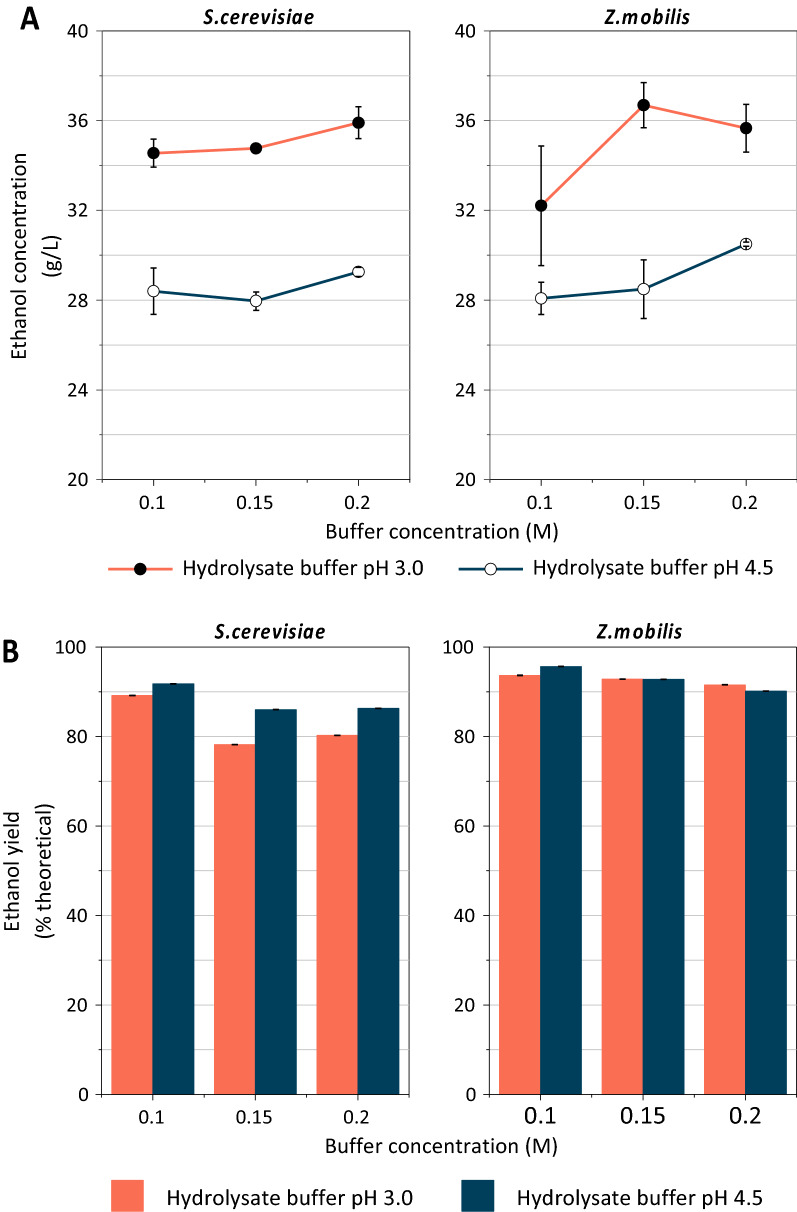
**A** Ethanol concentration was more affected by buffer pH (3.0 > 4.5) than concentration for both *S. cerevisiae* and *Z. mobilis* in 6% glucan loading hydrolysate. **B** Ethanol yield was not affected significantly by hydrolysate buffer pH. For both *S. cerevisiae* and *Z. mobilis*, the yields were higher at a lower buffer concentration for both cases. All hydrolysates were made from AFEX pretreated CS at 6% glucan loading. All fermentations were conducted at a pH of 5.8 ± 0.1. Values for all subfigures are reported as mean ± SD, *n* = 2

Since the addition of phosphate buffer could have downstream effects on the fermentation microbes, we next sought to determine the effect of buffer concentration and pH added during enzymatic hydrolysis on subsequent microbial fermentation. First, the high solids loading hydrolysates were adjusted to pH 5.8 ± 0.1, which was suitable for fermentation by both *S. cerevisiae* and *Z. mobilis*. Engineered *S. cerevisiae* and *Z. mobilis* strains were then inoculated into sealed serum bottles containing various hydrolysates, grown at 30 °C for two days, and sampled for final ethanol and sugar concentrations. The ethanol concentrations at the end of fermentation were not dependent on buffer concentration, which for the same buffer pH were very similar (Fig. 2A). In contrast, ethanol concentrations were consistently higher for a buffer pH of 3.0 than 4.5 for both *S. cerevisiae* and *Z. mobilis* (Fig. 2A). For *Z. mobilis,* this appears to be entirely related to increased sugar concentrations in the hydrolysates, as the ethanol yields were very similar across all buffers (Fig. 2B). In contrast, the ethanol yields obtained for *S. cerevisiae* were slightly higher for the hydrolysate generated using the pH 4.5 buffer (Fig. 2B), indicating that though less ethanol was produced (Fig. 2A), the yeast was more efficient than *Z. mobilis* at converting sugars to ethanol.

### Sugar yields decline with increasing solids loading due to low water availability for liquefaction

**Fig. 3:**
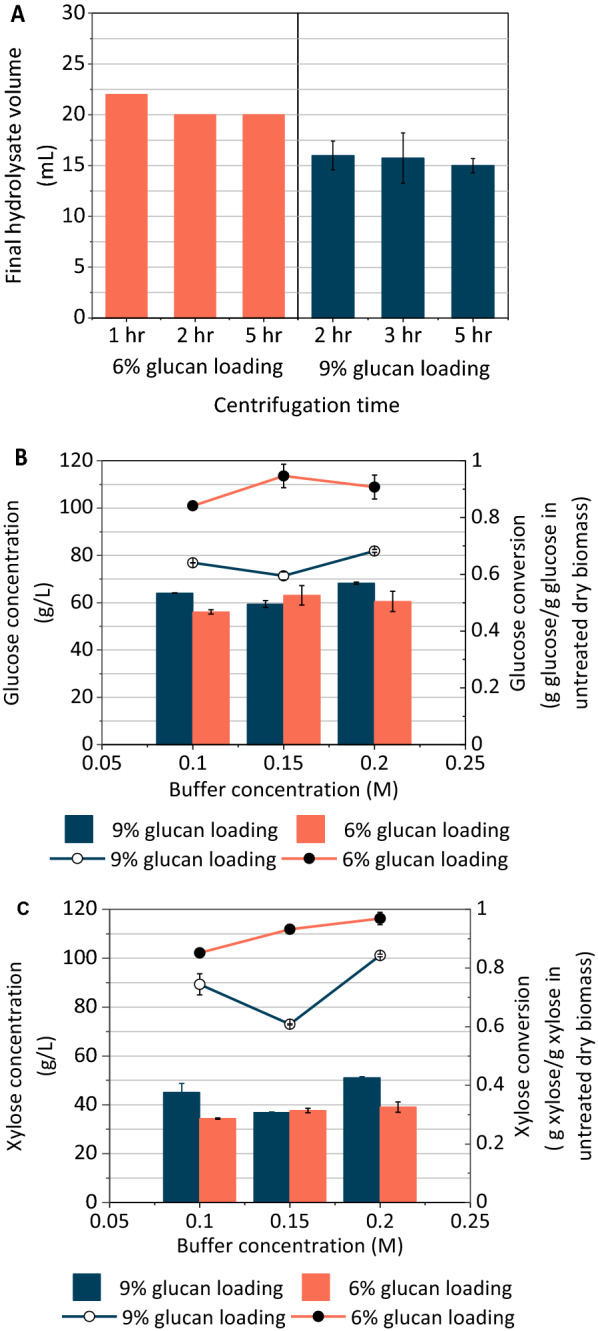
**A** 6% glucan loading produced higher volume of hydrolysate than 9% glucan loading for all centrifugation times. Also, the volume of hydrolysate was not affected by the centrifugation times. **B** Although the glucose concentrations were similar for both solids loading conditions, the glucose conversion was higher for 6% glucan loading than 9% glucan loading for all buffer concentrations. **C** Xylose conversion was higher for 6% glucan loading than 9% glucan loading for all buffer concentrations. Values for all subfigures are reported as mean ± SD, *n* = 2

Enzymatic hydrolysis was carried out on AFEX pretreated corn stover at 6% and 9% glucan loading (19% and 28% w/w solids loading, respectively) in roller bottles to evaluate the effect of solids loading on hydrolysate characteristics and optimum processing conditions. For the same buffer concentrations and buffer pH, 6% glucan loading showed more consistent liquefaction (Fig. 3A), and higher glucose and xylose conversions compared to 9% glucan loading (Fig. 3B and 3C). The glucose and xylose conversion were about 36% higher for 6% glucan loading compared to 9% glucan loading. The highest sugar conversions were obtained using the 0.2 M, pH 3.0 buffer for both solids loadings, with the exception of the 6% glucan hydrolysate, where glucose conversion was highest for the 0.15 M, pH 3.0 buffer (Fig. 3). An additional 5 mL was recovered from 6% glucan loading hydrolysates compared to the higher solids loading, though the volume did not vary significantly for the same solid loading across the reported centrifugation times. The 9% glucan loading samples were unable to be fully filtered by the dual stage filtration (0.5 μm pre-filtration followed by 0.22 μm sterile filtration) for centrifugation times less than 2 h, which is the reason for the difference in centrifugation times between the two solids loadings (Fig. 3A).

### Enzymatic hydrolysis in roller bottles led to greater liquefaction and higher sugar yields compared to shake flasks

**Fig. 4:**
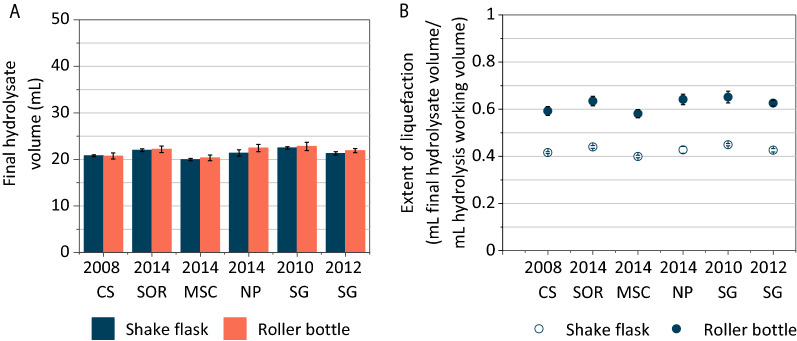
Roller bottle enzymatic hydrolysis achieves better liquefaction than the shake flask method, with equivalent final hydrolysate volumes despite different starting hydrolysis volumes. **A** Final hydrolysate volumes are similar for roller bottle and shake flask hydrolysis. **B** Extent of liquefaction is greater for roller bottle enzymatic hydrolysis. CS: corn stover; SOR: sorghum; MSC: miscanthus; NP: native prairie; SG: switchgrass. Numbers refer to the biomass harvest year. Values for all subfigures are reported as mean ± SD, *n* = 2. Values for all subfigures are reported as mean ± SD, *n* = 2

In order to validate the roller bottle enzymatic hydrolysis method, it was compared to enzymatic hydrolysis in shake flasks for a variety of herbaceous feedstocks (corn stover, switchgrass, sorghum, miscanthus, and native prairie). Roller bottle and shake flask experiments were conducted using the same conditions used previously at 6% glucan loading (15–22% w/w solids loading, depending on the feedstock). However, shake flasks had a greater working volume (50 mL in shake flask experiments compared to 35 mL for roller bottle experiments). In spite of this, the final hydrolysate volumes for the shake flask and roller bottle experiments were similar (Fig. 4A) and the extent of liquefaction, which is the ratio of final hydrolysate volume and working volume (Fig. 4B), was 42–47% higher for the roller bottle method for all feedstocks tested. A previously developed scalable roller bottle method for 20% (w/w) solids loading concluded that gravitational tumbling overcame the important bottlenecks of improper mixing and high viscosity when compared to the shake flask method with intermittent hand mixing [25], which agrees with our results.

**Fig. 5:**
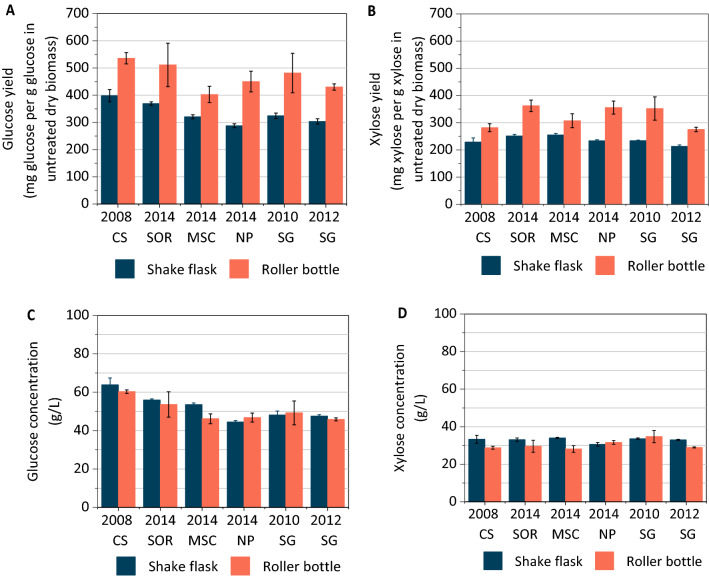
**A** Glucose yield was higher for the roller bottle hydrolysates than the shake flask hydrolysates for all the feedstocks. **B** Xylose yield was higher for the roller bottle hydrolysates than the shake flask hydrolysates. **C** Hydrolysate glucose concentration was similar for both roller bottle and shake flask samples. **D** Hydrolysate xylose concentration was similar for both roller bottle and shake flask samples. CS: corn stover; SOR: sorghum; MSC: miscanthus; NP: native prairie; SG: switchgrass. Numbers refer to the biomass harvest year. Values for all subfigures are reported as mean ± SD, *n* = 2. Values for all subfigures are reported as mean ± SD, *n* = 2

As expected from the greater extent of liquefaction, the glucose and xylose yields for the hydrolysates from the roller bottle method were ~ 25–50% higher than the shake flask method for all the AFEX pretreated feedstocks (Fig. 5A and B), though the hydrolysate sugar concentrations were similar for both methods (Fig. 5C and D). This indicates that although both methods seem to provide hydrolysate of similar quality from the same feedstock for fermentation, the roller bottle system facilitates greater conversion in the same amount of time and because of this generates greater usable hydrolysate volumes.

**Fig. 6:**
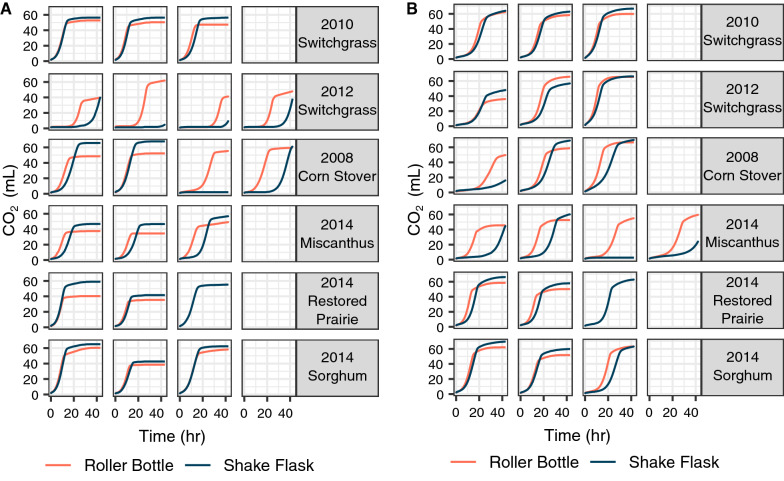
CO_2_ production by **A** *S. cerevisiae* Y945 and **B** *Z. mobilis* 2032 show some feedstock-specific differences during fermentation of hydrolysates generated in roller bottles or shake flasks. For each feedstock, columns represent separate paired replicates, where the two fermentations (shake flask and roller bottle system) were run simultaneously. (Plots in the same column across feedstocks were not necessarily run in the same batch of fermentations.)

**Fig. 7:**
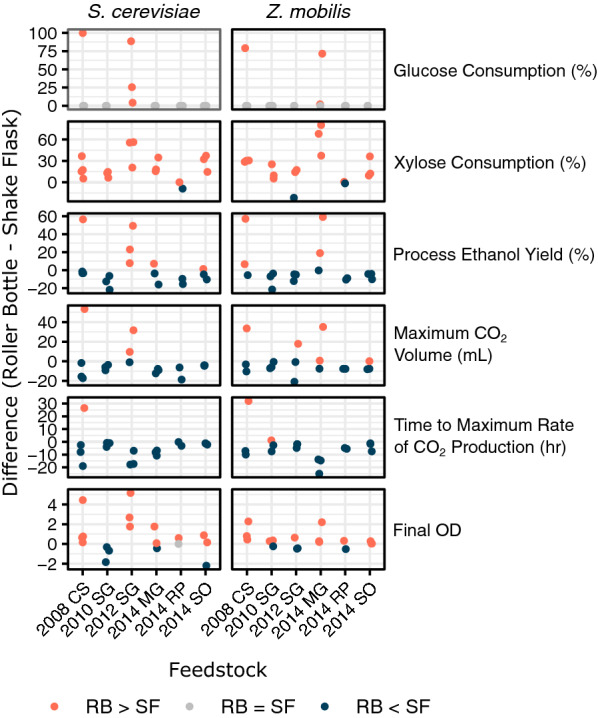
Max CO_2_ volume is correlated with the final volume of ethanol produced during fermentation for both *S. cerevisiae* Y945 and *Z. mobilis* ZM2032. Samples are 6% glucan loading hydrolysates produced using the roller bottle and shake flasks from all five grass feedstocks that had been pretreated in the 5-gallon reactor (2008 corn stover, 2010 and 2012 switchgrass, and 2014 miscanthus, native prairie, and sorghum)

We next compared the diverse hydrolysates generated by the roller bottle and shake flask hydrolysis in microbial fermentation experiments. Standard flask fermentations with yeast or bacteria typically require 10–20 mL of medium because a significant amount of culture volume is depleted from cell density (e.g*.*, OD_600_ measurements) and extracellular metabolite (e.g*.*, ethanol titer as determined by HPLC-RID) sampling. Since we had obtained only 20–25 mL of hydrolysate from both roller bottle and shake flask protocols, we investigated alternative methods of fermentation that utilize low (5 mL or less) volumes of hydrolysates, which will allow the remaining volume to be used for additional studies. Since CO_2_ is formed as a byproduct during the anaerobic fermentation of glucose to ethanol (C_6_H_12_O_6_ → 2 C_2_H_5_OH + 2CO_2_ + 2ATP), others have monitored fermentative CO_2_ production from the vessel headspace as a proxy for ethanol production [38, 39]. We developed a protocol that employs a commercial respirometer system that measures and records CO_2_ production in real time. Serum bottles containing 5 mL of paired hydrolysates generated from roller bottles or shake flasks were inoculated with yeast *S. cerevisiae* or bacteria *Z. mobilis.* The serum bottles were connected to a commercially available respirometer system that measures CO_2_ production by tracking disruption of a laser beam by bubbles released from the flasks. CO_2_ production was measured for approximately 48 h, at the end of which time final cell density and extracellular metabolite samples were taken for analysis. The data from the paired samples were subtracted to give an idea of general trends in fermentation performance between the two experimental methods. Interestingly, only the shake flask experiments had significantly inhibited fermentations that did not achieve maximum CO_2_ production by the end of the ~ 40 h fermentation period (Fig. 6). This is indicated by the positive data points for the roller bottle glucose consumption (Fig. 7). These values were high because the shake flask experiments for these paired samples had incomplete glucose consumption after ~ 40 h, while for all other experiments, 100% of the glucose was consumed (Additional file 1: Table S2 and S3).

In general, xylose consumption and final OD tended to be higher in fermentations with roller bottle hydrolysates, while process ethanol yield (amount of ethanol produced with respect to the theoretical maximum based on hydrolysate composition), maximum CO_2_ volume, and time to maximum rate of CO_2_ production were higher for shake flask experiments. The time to maximum rate of CO_2_ production gives an indication of delay in fermentation, with longer times for the shake flask experiments indicating slower and more inhibited fermentations. The few samples that did not follow these trends (the positive values in the respective plots in Fig. 7) were generally for the severely inhibited shake flask fermentations.

### The field-to-fuel platform successfully replicates fermentation results observed in larger-scale experiments

**Fig. 8:**
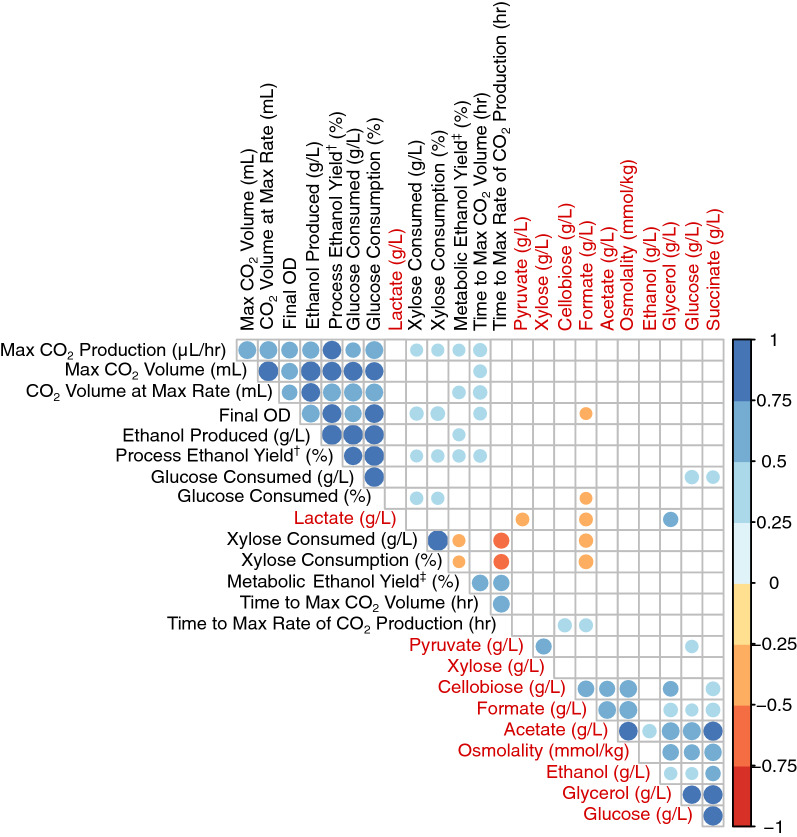
Clustered correlation matrix for *S. cerevisiae* Y945 fermentation data (black labels) and hydrolysate composition (red text labels). The plot was generated using the corrplot package in R with hclust (hierarchical clustering order). The size and color correspond to the direction and magnitude of the correlation. Correlations that were insignificant (*p* > 0.05) were not plotted. ^†^The process ethanol yield is the ratio of sugars initially present in the hydrolysate (glucose and xylose) to ethanol produced assuming 0.51 g ethanol/g sugars as the theoretical maximum. ^‡^The metabolic yield is the ratio of sugars (glucose and xylose) consumed during fermentation to ethanol produced assuming 0.51 g ethanol/g sugars as the theoretical maximum

Because hydrolysate composition is related to fermentation performance, we determined correlations between key fermentation results and hydrolysate sugars, alcohols, and organic acids (Fig. 8 and Additional file 1: Figure S2). For the most part, the compounds quantified in the hydrolysates were not correlated with key fermentation metrics, for either *S. cerevisiae* (Fig. 8) or *Z. mobilis* (Additional file 1: Figure S2) fermentations. Fermentation data tended to cluster together, and hydrolysate composition data tended to cluster together in the correlation plots. For the hydrolysate composition data, acetate, glucose, glycerol, and succinate concentrations were positively correlated across all feedstocks. The fermentation data showed strong positive correlations between glucose consumption (titer and percentage basis), process ethanol yield (amount of ethanol generated compared to the theoretical maximum), total ethanol produced, final cell density, maximum CO_2_ volume, and maximum rate of CO_2_ production.

**Fig. 9:**
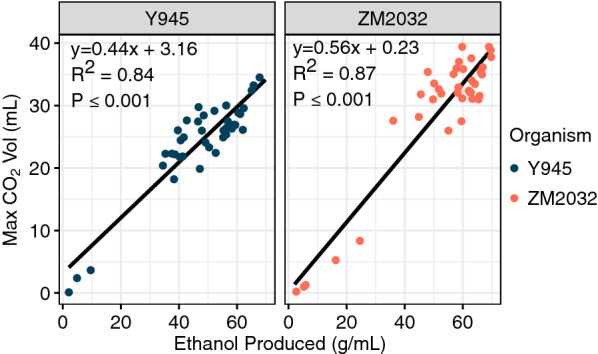
Max CO_2_ volume is correlated with the final volume of ethanol produced during fermentation for both *S. cerevisiae* Y945 and *Z. mobilis* ZM2032. Samples are 6% glucan loading hydrolysates produced using the roller bottle and shake flasks from all five grass feedstocks that had been pretreated in the 5-gallon reactor (2008 corn stover, 2010 and 2012 switchgrass, and 2014 miscanthus, native prairie, and sorghum)

Because the respirometer system measures CO_2_ volume as a proxy for ethanol yield, the strong correlation between the two values lends support for the utility of the method. When the two values are plotted, there is a strong positive correlation between the maximum CO_2_ volume and ethanol yield (*p* ≤ 0.01, *R*^2^ = 0.84 or 0.87); however, for productive fermentations, there is a large amount of scatter for the correlation between max CO_2_ volume and ethanol concentration for the 6% glucan loading hydrolysates (Fig. 9). In spite of this, unusually low final CO_2_ volumes corresponded very closely with low ethanol concentrations in the fermentation media (Fig. 9), indicating that CO_2_ is an adequate surrogate for ethanol production when used to identify severely inhibited fermentations.

**Fig. 10:**
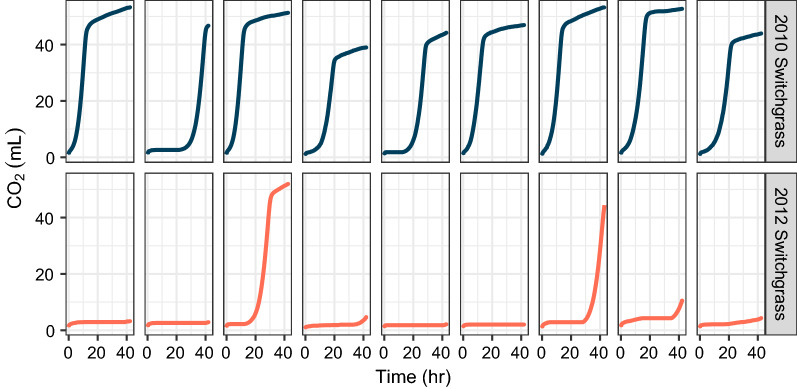
Saccharomyces cerevisiae grown in drought year (2012) switchgrass hydrolysates showed significantly reduced or delayed CO_2_ production compared to when it was grown in 2010 switchgrass hydrolysates from a year with normal precipitation. Each graph represents a separate batch of hydrolysate and each column represents paired replicates that were fermented in the same respirometer experiment

We also used the pipeline to process two feedstocks, switchgrass grown in a drought year (2012) and switchgrass grown in a year with normal precipitation (2010), which had previously shown strongly divergent yeast fermentation performance. These materials were pretreated in the custom AFEX reactors, hydrolyzed in the roller bottle system at 7% glucan loading (the same loading as previously published [11]), and conducted fermentation in the respirometer. Based on these experiments, switchgrass grown in a drought year (2012) was significantly more inhibitory to yeast fermentation compared to switchgrass grown in a normal year (2010) (Fig. 10). Although not all fermentations showed the complete inhibition of growth in the drought year switchgrass that was previously observed [11], all of the drought year samples had either significantly reduced or delayed CO_2_ production compared to their paired fermentation sample from the year with normal precipitation.

## Discussion

Feedstocks across multiple plots and locations need to be studied to correlate the effects of environmental factors on biofuel production. The high throughput required for such studies led to the need for a field-to-fuel research pipeline, combining pretreatment, enzymatic hydrolysis, and fermentation. For biomass pretreatment, we previously designed and constructed a customized system that can AFEX pretreat as little as 25 g of biomass [32]. The ability to process biomass in smaller quantities under process conditions that are similar to the previously used Parr reactor system [11] makes the custom reactor suitable for the field-to-fuel research pipeline. The glucose and xylose concentrations after enzymatic hydrolysis for the AFEX pretreated biomass using the customized system are comparable to the results obtained for the biomass processed in the 5-gallon Parr reactor system [12, 32]. The 2010 and 2012 harvested switchgrass that were used for the final validation (Fig. 10) were pretreated in these custom AFEX reactors.

While several methods for enzymatic hydrolysis have been developed, they are less amenable for high solids loading with limited biomass quantity. High viscosity, product inhibition, low water availability, accumulation of oligosaccharides, and inhibition of enzyme adsorption are some of the most important reasons for low sugar conversion during enzymatic hydrolysis of lignocellulosic biomass at high solids loading, especially in stirred tank reactors and laboratory-scale shake flask reactors [22, 23, 40]. Horizontal bioreactors have achieved faster liquefaction through effective mixing of substrate and enzymes, which was evident through the drastic decrease in viscosity at high solids loading when compared to shake flask [41, 42]. A 2 L horizontal bioreactor designed for high solids loadings has shown enhanced biomass liquefaction and glucose yields up to 97.99% for 25% and 30% w/v solids loading, which is comparable to our results 94.6% glucose yield obtained for the roller bottle method at 6% glucan loading [24]. Lower efficiency enzymatic hydrolysis leads to lower volumes of hydrolysate and final products after microbial fermentation [43]. Liquefaction during high solids enzymatic saccharification has been a predominant method to evaluate the deconstruction of lignocellulosic biomass. Horizontal mixing in the roller bottle method has been shown to overcome the liquefaction problem in shake flasks at high solids loading, increasing sugar conversion and liquefaction, while generating hydrolysates with similar sugar composition. A previously studied 1.7 L horizontal rotating reactor at 25% w/w solids loading for steam pretreated corn stover was able to provide 20% higher saccharification when compared to a vertical stirred tank reactor [26]. In our study, liquefaction was about 45% higher for the roller bottle method than the shake flask method, consistently producing higher volumes of fermentable hydrolysates from AFEX pretreated biomass. One alternative route for improved hydrolysis performance in shake flasks is to load samples in fed batch. However, our process follows a strict protocol to maintain aseptic conditions and prevent microbial contamination, as has been observed to be an issue in other studies [44]. Fed-batch addition of biomass is incompatible with these methods, and for this reason, we were not able to use a fed-batch approach.

The effect of solids loading on enzymatic hydrolysis on the hydrolysate composition was compared for corn stover at 6% and 9% glucan loading (19% and 28% w/w solids loading) during process optimization. Our results (Fig. 3A and B) are consistent with a previous work on dilute acid pretreated corn stover loaded at 5%, 10%, and 15% w/w solids loading, where the highest glucan conversion was observed for 5% solids loading [30]. Similarly, for an increase in solids loading from 2 to 5% for steam pretreated softwood resulted in a 16% decreasing of carbohydrate conversion [45]. Glucose conversions are known to decrease with increasing solids loading [14]. This is attributed to both end product inhibition of enzymes and accumulation of oligosaccharides at high solids loading [28, 40]. We observed that liquefaction and sugar release were higher for 6% glucan loading compared to 9% glucan loading. The availability of initial free liquid at the beginning of the enzymatic hydrolysis could account for the better liquefaction at 6% glucan loading. For 9% glucan loading, the water added to the pretreated biomass was absorbed completely by the biomass and consequently, no initial free liquid was available, which may have caused slower deconstruction of the pretreated biomass, as seen elsewhere [16, 17].

**Fig. 11:**
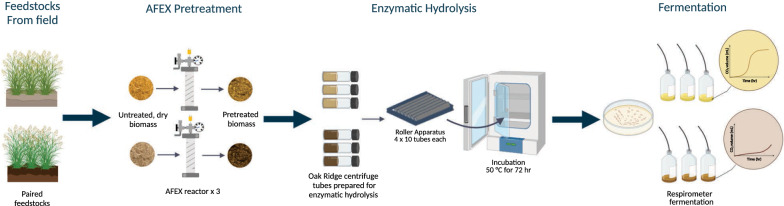
Process flowchart for the field-to-fuel platform including small-scale AFEX pretreatment, roller bottle enzymatic hydrolysis, and respirometer fermentation using *S. cerevisiae* and *Z. mobilis*. Created with Biorender.com

A major goal of our platform (Fig. 11) is to rapidly compare environmentally challenged feedstock samples to determine the impact on fermentation compared to the general population, and use this to identify contributing biomass environmental, agronomic, or genetic factors. While ethanol is the desired product, it is challenging to monitor ethanol concentrations at informative time resolution from low culture volumes. Using CO_2_ production as a surrogate for ethanol production is one way to monitor fermentation progress real time, while avoiding issues with sampling and disturbing the fermentation process. Our research pipeline showed some correlation between final ethanol titer and maximum CO_2_ production in fermentations of multiple feedstocks, and accurately represents differences in growth and fermentation across paired samples, particularly when inhibitory hydrolysates are compared (e.g*.*, 2012 drought versus 2010 normal year switchgrass). When comparing the fermentability of hydrolysates generated using the roller bottle and shake flask methods, in general, the shake flask hydrolysates were more inhibited based on the longer time required to reach exponential CO_2_ production and lower final cell density, and some samples were unable to fully utilize all of the glucose by the end of the ~ 40 h fermentation period. Though slower, the fermentations using the shake flask hydrolysates tended to have greater CO_2_ and ethanol production compared to their paired roller bottle hydrolysates. Interestingly, the xylose consumption was also lower in the shake flask hydrolysates, which is opposite the trend observed for diverse feedstocks, where greater xylose consumption tends to correlate with a higher process ethanol yield [12, 46]. The process ethanol yield for diverse feedstocks were similar with a few exceptions for the roller bottle hydrolysates, which is the same trend as observed in the previous study, in which the hydrolysates were generated in a 3 L Applikon *ez*-control bioreactor system (Applikon Biotechnology, Foster City, CA, USA) [12]. Ultimately, by using our small-scale pretreatment, roller bottle enzymatic hydrolysis at optimal conditions, and monitoring CO_2_ production during fermentation, we were able to replicate the results previously observed [11] showing significant, replicated inhibition of drought year switchgrass with respect to the control switchgrass. Inhibitory hydrolysates could be targeted for more detailed analysis, such as chemical genomics studies [47, 48] or scaled-up experiments in bioreactors. Therefore, the field-to-fuel research pipeline can be used to compare multiple samples from across the field for statistical confidence.

## Conclusion

This study has demonstrated that the roller bottle system is able to better overcome the major bottlenecks of poor mixing, inadequate availability of water, and viscous nature of pretreated biomass compared to the shake flask method, for a variety of grass-based AFEX-pretreated feedstocks [15]. When the entire field-to-fuel platform was used, combining moderate-scale pretreatment, roller bottle enzymatic hydrolysis, and respirometer fermentation, we were able to replicate the fermentation differences from limited volumes of hydrolysates from 2012 switchgrass grown in a drought year compared to 2010 switchgrass grown under normal precipitation. This method can be utilized to compare an array of feedstocks and different process conditions.

## Materials and methods

### Biomass growth, harvest, and processing

The biomass samples used for the experiments in this study (corn stover, switchgrass, sorghum, restored prairie, and miscanthus) were cultivated at the DOE-Great Lakes Bioenergy Research Center’s (GLBRC) Biofuel Cropping Systems Experiments (BCSE) located at the Arlington Agricultural Research Station in southcentral Wisconsin, USA (ARL, 43$^\circ$ 17′ 45″ N, 89$^\circ$22′ 48″ W, 315 m a.s.l) and the W.K. Kellogg Biological Station in southwest Michigan, USA (KBS, 42$^\circ$ 23′ 47″ N, 85$^\circ$ 22′ 26″ W, 288 m a.s.l) [49, 50]. The mean annual temperature and precipitation were 6.9 $^\circ \mathrm{C}$ and 869 mm, respectively. The soil type is Plano silt loam, which is fine-silty, mixed, super-active, mesic Typic Argiudoll, well drained. Mollisol developed over glacial till and formed under tallgrass prairie. Switchgrass (SG) was sourced from ARL-346 in both 2010 and 2012. Sorghum (SOR), Miscanthus (MSC), and Restored Prairie (RP) were sourced from ARL-AUX TRIAL, ARLG6R5, and ARLG5R4 in 2014. Corn stover (CS) was sourced from ARL570 in 2008. Field plots (28 m × 40 m) were harvested and chopped into a wagon. When the wagons were unloaded, a representative 25 kg sample was collected. The harvested plant materials were dried in a 60 °C oven, milled using a Christy Turner mill (Christy Turner Ltd. Suffolk, UK), and then mixed by hand to ensure homogeneity before being packaged in sealed plastic bags until use. The composition testing conducted previously across multiple bags and feedstocks have not shown significant difference in biomass composition, the data for which have not been published. We used corn stover samples for the optimization of the high solids loading roller bottle enzymatic hydrolysis method. The switchgrass (SG), sorghum (SOR), miscanthus (MSC), and restored prairie (RP) samples, which are mentioned above, were used to confirm the effectiveness of the method on a variety of grass feedstocks.

### Cell wall and bulk chemical composition of biomass

The samples were milled before the analysis using a Cyclotec™ mill (Foss, Denmark), equipped with a 2 mm screen. The composition of the bulk biomass (Additional file 1: Table S1) was determined using the standard method described by the NREL laboratory analytical procedures for composition analysis of biomass [51]. All composition experiments were performed in triplicate.

### Ammonia fiber expansion (AFEX) pretreatment

The corn stover, switchgrass, sorghum, restored prairie, and miscanthus samples were pretreated using ammonia fiber expansion (AFEX) pretreatment. The pretreatment experiments were carried out in a 3.8 L high-pressure Parr reactor (Parr Instrument Co. Moline, IL, USA), which was placed inside a walk-in fume hood. Dry biomass mixed with water (0.6 g H_2_O/g dry biomass) was loaded into the Parr reactor and sealed. The sealed reactor was charged with nitrogen to 60 psi. The reactor was then preheated to suitable temperatures according to the type of biomass. Liquid ammonia, at a loading of 2 g NH_3_/g dry biomass, was added to the biomass using a LEWA EK_1_ metering pump (Leonberg, Germany) [11]. After ammonia loading, the reactor temperature was increased to the set point within 5 min and then maintained at the set point temperature for the 30 min residence time. At the completion of the reaction, the ammonia was vented out from the reactor inside the walk-in fume hood. The pretreated biomass was then dried in a custom fume-vented drying box. The dried AFEX pretreated biomass (< 12% moisture content on a total weight basis) was packed into sterilized bags and stored at room temperature until it was used [44].

The 2010 and 2012 harvested switchgrass that were used for the field-to-fuel process validation studies were pretreated in custom AFEX reactors, as described previously [32]. In brief, 25 g of untreated biomass (dry weight basis) was mixed with water (0.6 g H_2_O per g dry biomass) and loaded into the custom pretreatment reactors. The reactor was preheated to 60 °C, and then ammonia was added using a high-pressure ammonia syringe pump (Harvard Apparatus 70-3311) equipped with a 100 mL stainless steel syringe to achieve a loading of 2 g NH_3_ per g dry biomass. The reactor was heated to 120 °C and maintained at the set point until 30 min after ammonia addition, at which point the reactor was vented, cooled, and unloaded. The pretreated biomass was dried in a custom drying box and stored in plastic bags at room temperature until used.

### High solids roller bottle enzymatic hydrolysis

High solids roller bottle enzymatic hydrolysis experiments were optimized using AFEX pretreated corn stover at 6% and 9% glucan loading (g glucan/mL) by adjusting the following conditions: (1) phosphate buffer pH, (2) phosphate buffer concentration, and (3) centrifugation time. All hydrolysate samples were loaded in 85 mL Nalgene Oak Ridge centrifuge tubes, with a final working volume of 35 mL. The biomass was autoclaved at 121 °C for 20 min to prevent microbial contamination. After the autoclave step, a designated volume of phosphate buffer (0.05 M, 0.1 M, 1.5 M or 2.0 M; and pH 3.0 or pH 4.5), consisting of monobasic and dibasic potassium phosphate, enzymes, and makeup water to account for the amount lost during autoclaving, was added to the centrifuge tubes inside a laminar flow hood. The centrifuge tubes were then sealed with caps, which had been sterilized with 10 vol% bleach solution prior to the experiment. Novozyme 22257 cellulase and Novozyme 22244 hemicellulase (Novozymes, Franklinton, NC, USA) were desalted using a disposable desalting column (Disposable PD-10 Desalting Columns, Cytiva, VWR Catalog. No. 95017-001) and analyzed for protein content using the Pierce™ BCA Protein Assay Kit (Pierce Biotechnology). Enzymes were loaded at 28 mg protein/g glucan, consisting of 70% cellulase and 30% hemicellulase (v/v). The sealed centrifuge tubes were placed on a laboratory-scale bottle roller (Low Profile Roller, IBI Scientific, Low Profile Roller Lab Start-Up Kit) at 20 rpm inside a static incubator (VWR symphony™, 414004-626, Low temp./BOD Incubator) set at 50 °C. A single static roller can accommodate up to 10 centrifuge tubes. After 72 h of enzymatic hydrolysis, the samples were centrifuged for various times at 12,000 rpm (18,500×*g*) and 4 °C in a benchtop laboratory-scale centrifuge (Eppendorf Benchtop 5804R Centrifuge). At a constant rotation speed of 12,000 rpm (18,500×*g*), 6% glucan loading hydrolysates (0.1 M phosphate, pH 3.0) were centrifuged for 1 h, 2 h, or 5 h. The 9% glucan loading hydrolysates were centrifuged at 12,000 rpm (18,500×*g*) for 2 h, 3 h, or 5 h. The final pH of the supernatant was recorded for all the samples and then adjusted to a suitable pH for fermentation by either *Saccharomyces cerevisiae* or *Zymomonas mobilis,* 5.8 ± 0.1, using 12 M HCl or 10 M NaOH. The pH-adjusted hydrolysates were pre-filtered through 0.5 µm glass fiber filter paper (Metrigard*®*, 47 mm, Pall, VWR Catalog. No. 28150-371) in a 4.7-cm-diameter Buchner funnel. This filtrate was then sterile filtered using a 0.22 µm, 50 mL Autofil sterile filtration system. The hydrolysate samples were collected in sterile polypropylene centrifuge tubes and stored at 4 °C until shipped on ice for subsequent fermentation experiments and characterization.

The effectiveness of the method was then tested on 6% glucan loading AFEX treated switchgrass from two different years of harvest (2010 and 2012), and sorghum, miscanthus, and restored prairie harvested in 2014 based on the optimized parameters for phosphate buffer pH (3.0) and phosphate buffer concentration (0.1 M). Centrifugation time of 1 h was sufficient for corn stover harvested in 2008, whereas 3 h centrifugation was required for the other feedstocks for proper solid–liquid separation. Therefore, the centrifugation time for the final method was 3 h. For the field-to-fuel validation studies, 2010 and 2012 switchgrass pretreated in the smaller-scale custom reactors were processed using the same method, but at 7% glucan loading to match the conditions used in previous studies [11, 44]. All other hydrolysis conditions were identical.

### High solids loading enzymatic hydrolysis–shake flask method

The roller bottle enzymatic hydrolysis method was compared to the conventional shake flask method in batch mode. The experiments were conducted at 6% glucan loading, with 0.1 M phosphate buffer at pH 3.0 for 72 h on AFEX pretreated corn stover (CS), sorghum (SOR), switchgrass (SG), miscanthus (MSC), and restored prairie (RP). The working volume for the flask method was 50 mL, as opposed to the 35 mL used in the roller bottle system. AFEX pretreated samples were added to previously autoclaved 100 mL Erlenmeyer flasks. The biomass was autoclaved at 121 °C for 20 min to prevent microbial contamination and match the roller bottle method. After the autoclave step, a designated volume of 0.1 M phosphate buffer (pH 3.0) consisting of monobasic and dibasic potassium phosphate, enzymes (28 mg protein per g glucan—70% cellulase and 30% hemicellulase), and makeup water to account for the amount lost during autoclaving was added to the Erlenmeyer flasks inside a laminar flow hood. The Erlenmeyer flasks were then sealed with 27-mm-diameter rubber stoppers, which had been autoclaved prior to the experiment. The sealed Erlenmeyer flasks were placed inside a shaker incubator (New Brunswick™ Excella® E25) at 150 rpm set at 50 °C. After 72 h of enzymatic hydrolysis, the samples were centrifuged in 85 mL Nalgene Oak Ridge centrifuge tubes for 3 h at 12,000 rpm (18,500×*g*) and 4 °C in a benchtop laboratory-scale centrifuge (Eppendorf Benchtop 5804R Centrifuge). The final pH of the supernatant was recorded for all the samples. The pH was then adjusted to the optimum pH for fermentation by either *Saccharomyces cerevisiae* or *Zymomonas mobilis,* 5.8 ± 0.1, using 12 M HCl or 10 M NaOH to ensure adequate pH for fermentation. The pH-adjusted hydrolysates were pre-filtered through 0.5 µm glass fiber filter paper (Metrigard*®*, 47 mm, Pall, VWR Catalog. No. 28150-371,) in a 4.7-cm-diameter Buchner funnel. This filtrate was then sterile filtered using a 0.22 µm, 50 mL Autofil sterile filtration system. The hydrolysate samples were collected in sterile polypropylene centrifuge tubes and stored at 4 °C until shipped on ice for subsequent fermentation experiments and characterization. The composition of glucose, xylose, and other hydrolysate end products (Additional file 1: Figure S1) was analyzed using HPLC-RID as described previously [52].

### Fermentation

For the fermentation experiments, 5 mL of each hydrolysate was pipetted into sterile serum bottles and degassed overnight in an anaerobic chamber. A culture of either *Z. mobilis* ZM2032 [53] or *S. cerevisiae* GLBRCY945 [54] was grown overnight and diluted into anaerobic media the day of the experiment. Once the cultures reached logarithmic growth, they were centrifuged, and cell pellets were resuspended with synthetic medium [44], and inoculated into 60 mL Wheaton serum bottles. The serum bottles were capped with airtight Chemglass Life Sciences Blue Butyl, 20 mm rubber caps and placed on a 120 rpm shaker in a 30 °C environmental growth chamber. The cultures were attached to respirometer cartridges using BD PrecisionGlide 23GX1 (0.6 mm × 25 mm) sterile needles inserted into the serum bottle caps. The respirometer (AER-800; Challenge Technology; Springdale, AR, USA) measured the volume of gas produced by the growing culture. Each experiment was run for 48 h, unless stated otherwise. Supernatants from post-fermentation cultures were analyzed by high-performance liquid chromatography (HPLC) and refractive index detection (RID) for sugar and ethanol concentrations [52]. Final cell density (OD_600_) measurements were made with a Beckman DU720 spectrophotometer.

### Calculations

#### Extent of liquefaction

The extent of  hydrolysate liquefaction was calculated as the volume of liquid recovered by centrifugation and filtration as a proportion of the hydrolysis working volume (total volume of solids, liquid, etc. in the vessel) [43],

$$Extent\, of\, liquefaction = \frac{Volume \,of \,supernatant \,following \,centrifugation \,and filtration}{{Initial \,working \,volume \,of \,the \,sample}}$$

#### Glucan conversion

$$\begin{gathered} Glucan \,conversion\, \left( {{\text{g}} \,glucose \,released \,per \,{\text{g}} \,glucose \,in \,untreated \,dry \,biomass} \right) \hfill \\ \quad = \frac{{glucose \,measured \,by \,HPLC\,\left( {\frac{{\text{g}}}{{\text{L}}}} \right)}}{1000 \times glucan \,loading \% } \times \frac{162.14}{{180.16}} \hfill \\ \end{gathered}$$

where MW_glucose_  = (180.16 g/mol) and  MW_glucan_  = (162.14 g/mol).

#### Glucose yield

Glucose yield was calculated as described previously [55]

$$\begin{gathered} Glucose\, yield\, \left( {{\text{mg}}\, glucose \,released \,per\, {\text{g}}\, glucose \,in \,untreated \,dry \,biomass} \right) \hfill \\ \quad = \frac{{glucose \,measured \,by \,HPLC\left( {\frac{{\text{g}}}{{\text{L}}}} \right)}}{1000 \times glucan \,loading \% } \times \frac{162.14}{{180.16}} \times V_{a} \hfill \\ \end{gathered}$$

where MW_glucose_ = (180.16 g/mol) and MW_glucan_ = (162.14 g/mol) and *V*_*a*_ is the available volume of the hydrolysate.

#### Xylan conversion

$$\begin{gathered} Xylan\, conversion \,\left( {{\text{g}} \,xylose released \,per \,{\text{g}} \,xylose \,in \,untreated \,dry \,biomass} \right) \hfill \\ \quad = \frac{{xylose \,measured \,by \,HPLC\,\left( {\frac{{\text{g}}}{{\text{L}}}} \right)}}{1000 \times glucan \,loading \% } \times \frac{{\% glucan \,content \,per\, {\text{g}}\, untreated \,biomass}}{{\% xylan \,content \,per \,{\text{g}}\, untreated \,biomass}}\user2{ } \times \frac{132}{{150}} \hfill \\ \end{gathered}$$

where MW_xylose_ = (150 g/mol) and MW_xylan_ = (132 g/mol).

#### Xylose yield

Xylose yield was calculated as follows:

$$\begin{gathered} Xylose yield \left( {\text{mg} \,xylose\, released \,per {\text{g}} xylose \,in \,untreated\, dry\, biomass} \right) \hfill \\ \quad = \frac{{xylose\, measured \,by\, HPLC\left( {\frac{{\text{g}}}{{\text{L}}}} \right)}}{1000 \times glucan \,loading \% } \times \frac{{\% glucan \,content \,per \,{\text{g}}\, untreated \,biomass}}{{\% xylan \,content \,per {\text{g}}\, untreated \,biomass}}\user2{ } \times \frac{132}{{150}} \times V_{a} \hfill \\ \end{gathered}$$

where MW_xylose_ = (150 g/mol) and MW_xylan_ = (132 g/mol) and *V*_*a*_ is the extent of liquefaction.

#### Ethanol yield

Ethanol yield was calculated as follows [56]

$$Ethanol yield \;\left( {\% \;of theoretical} \right) = \frac{{ethanol measured by HPLC\left( {\frac{{\text{g}}}{{\text{L}}}} \right)}}{{\left( {glucose\left( {\frac{{\text{g}}}{{\text{L}}}} \right) + xylose\left( {\frac{{\text{g}}}{{\text{L}}}} \right)} \right)consumed \times 0.51}} \times 100\%$$

where the theoretical maximum yield of ethanol from both glucose and xylose is 0.51 g ethanol produced per g sugar consumed.

## Supplementary Information

**Additional file 1: Table S1.** Untreated feedstock composition for different types of cellulosic biomass. **Figure S1.** Roller Bottle and shake flask hydrolysate composition. **Figure S2.** Clustered correlation matrix for Z. mobilis 2032 fermentation data and hydrolysate composition. **Table S2.** Summary of *Saccharomyces cerevisiae* Y945 fermentation results. **Table S3.** Summary of *Zymomonas mobilis* 2032 fermentation results.

## Data Availability

The datasets used and/or analyzed during the current study are available from the corresponding author on reasonable request.

## Abbreviations

CS: Corn stover
SOR: Sorghum
MSC: Miscanthus
NP: Native prairie
SG: Switchgrass
AFEX: Ammonia fiber expansion
