# Supplementary material for: A high solids field-to-fuel research pipeline to identify interactions between feedstocks and biofuel production
Source: Biotechnol Biofuels. 2021 Sep 10;14:179. doi: 10.1186/s13068-021-02033-6 (PMC8431876; doi:10.1186/s13068-021-02033-6)
Supplement: Supplementary file 1 — Additional file 1: Table S1. Untreated feedstock composition for different types of cellulosic biomass. Figure S1. Roller Bottle and shake flask hydrolysate composition. Figure S2. Clustered correlation matrix for Z. mobilis 2032 fermentation data and hydrolysate composition. Table S2. Summary of Saccharomyces cerevisiae Y945 fermentation results. Table S3. Summary of Zymomonas mobilis 2032 fermentation results. [file 13068_2021_2033_MOESM1_ESM.pdf]

Additional Information

A high solids field to fuel research pipeline to identify interactions between feedstocks and biofuel production

Meenaa Chandrasekar[1,2], Leela Joshi[1,2], Karleigh Krieg[1,2], Sarvada Chipkar[1,2], Emily Burke[1,2], Derek J Debrauske[3], Kurt D Thelen[4], Trey K Sato[3], Rebecca G Ong[1,2]

Affiliations

[1] DOE Great Lakes Bioenergy Research Center, Michigan Technological University, Houghton, MI, USA

[2] Department of Chemical Engineering, Michigan Technological University, Houghton, MI, USA

[3] DOE Great Lakes Bioenergy Research Center, Univ. of Wisconsin-Madison, USA

[4] DOE Great Lakes Bioenergy Research Center, Michigan State University, East Lansing, MI, USA

Contents

Table S1: Untreated feedstock composition for different types of cellulosic biomass.

Figure S1: Roller Bottle and shake flask hydrolysate composition.

Figure S2: Clustered correlation matrix for Z. mobilis 2032 fermentation data and hydrolysate composition.

Table S2: Summary of *Saccharomyces cerevisiae* Y945 fermentation results.

Table S3: Summary of *Zymomonas mobilis* 2032 fermentation results.

Table S1: Untreated feedstock composition for different types of cellulosic biomass.

Feedstock composition	2008 Corn stover (CS)	2014 Sorghum (SOR)	2010 Switchgrass (SG)	2012 Switchgrass (SG)	2014 Miscanthus (MSC)	2014 Restored prairie (RP)
Total structural sugars	59.15 ± 0.28	61.37 ± 0.15	60.41 ± 0.18	53.70 ± 0.39	65.99 ± 0.17	61.93 ± 0.51
Glucan	34.47 ± 0.16	37.19 ± 0.12	34.86 ± 0.11	30.35 ± 0.39	41.28 ± 0.11	36.97 ± 0.48
Xylan	20.17 ± 0.23	20.54 ± 0.06	21.60 ± 0.13	18.98 ± 0.04	21.39 ± 0.11	20.40 ± 0.15
Galactan	2.90 ± 0.03	0.91 ± 0.01	1.48 ± 0.03	1.62 ± 0.03	1.09 ± 0.03	1.45 ± 0.06
Arabinan	1.61 ± 0.04	1.83 ± 0.03	2.47 ± 0.03	2.76 ± 0.01	2.17 ± 0.05	2.05 ± 0.03
Mannan	NM*	0.9 ± 0.08	NM*	ND**	0.05 ± 0.0	1.06 ± 0.05
Acetyl groups	2.60 ± 0.10	2.50 ± 0.11	2.58 ± 0.11	2.15 ± 0.06	2.24 ± 0.03	2.63 ± 0.03
Lignin	13.79 ± 0.27	16.18 ± 0.17	18.59 ± 0.11	15.38 ± 0.06	20.13 ± 0.54	18.98 ± 0.34
Acid insoluble lignin (Klason lignin)	12.45 ± 0.05	15.21 ± 0.17	17.87 ± 0.10	14.31 ± 0.06	19.12 ± 0.54	17.86 ± 0.34
Acid soluble lignin	1.35 ± 0.26	0.97 ± 0.02	0.73 ± 0.03	1.07 ± 0.01	1.01± 0.01	1.12 ± 0.03
Protein	3.72 ± 0.03	2.87 ± 0.15	3.19 ± 0.09	4.65 ± 0.08	2.54 ± 0.09	3.19 ± 0.11
Ash	6.28 ± 0.17	5.27 ± 0.05	5.56 ± 0.16	5.47 ± 0.08	3.98 ± 0.09	5.45 ± 0.10
Total extractives	19.04 ± 0.16	18.53 ± 0.11	14.90 ± 0.26	22.14 ± 0.21	8.74 ± 0.07	11.34 ± 0.13

* NM= not measured
**ND= not detected

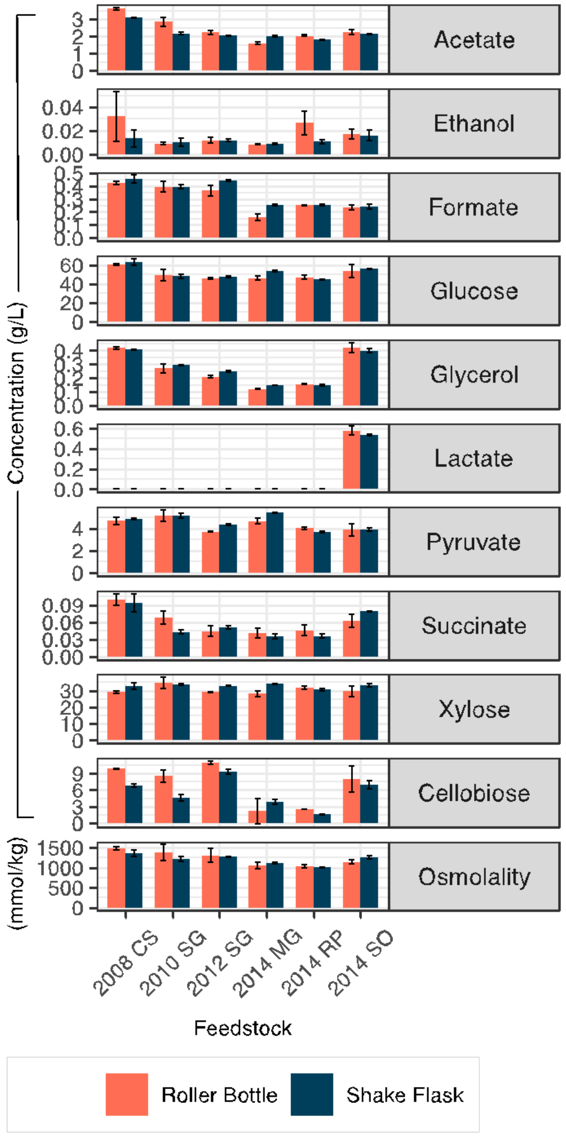

Figure S1)Roller Bottle and shake flask hydrolysate composition. Bars represent the average with error bars as ± standard deviation.

Table S2: Summary of *Saccharomyces cerevisiae* Y945 fermentation results. Values are reported as the mean ± s.d. (n=3 or 4).

	2008 CS	2010 SG	2012 SG	2014 MG	2014 RP	2014 SO
**Roller Bottle**						
Final OD	5.1 ± 0.4	4.1 ± 0.3	6.1 ± 0.5	5.3 ± 1.4	5.0 ± 0.0	5.4 ± 0.1
Glucose Consumption (%)	100 ± 0.0	100 ± 0	99.9 ± 0.3	100 ± 0	100 ± 0	100 ± 0
Xylose Consumption (%)	27.1 ± 2.4	38.2 ± 5.5	39.6 ± 14.2	44.6 ± 16.8	35.1 ± 2.5	51.1 ± 24.2
Glucose Consumption (g/L)	60.4 ± 0.8	49.2 ± 6.2	45.8 ± 0.6	46.2 ± 2.6	46.8 ± 2.3	53.6 ± 6.6
Xylose Consumption (g/L)	7.9 ± 0.8	13.3 ± 2.8	11.5 ± 4.2	12.4 ± 3.8	11.1 ± 1.1	15.3 ± 7.4
Ethanol Produced (g/L)	27.6 ± 1.4	21.9 ± 1.8	25.0 ± 2.1	22.3 ± 1.8	22.0 ± 0.4	25.8 ± 3.4
Process Ethanol Yield (%)[§]	60.7 ± 3.9	51.8 ± 9.3	65.4 ± 4.8	59.0 ± 8.4	55.1 ± 1.4	61.2 ± 10.3
Metabolic Ethanol Yield (%)[‡]	79.4 ± 5.0	69.8 ± 13.9	85.5 ± 3.2	74.5 ± 5.7	74.7 ± 3.1	74.2 ± 9.3
Maximum Rate of CO_2 Production (µL/hr)	1.6 ± 0.1	1.7 ± 0.2	2.0 ± 0.3	1.4 ± 0.1	1.7 ± 0.3	1.8 ± 0.3
Time to Maximum Rate of CO_2 Production (hr)	17.0 ± 7.2	10.2 ± 0.8	27.6 ± 5.0	10.9 ± 1.6	9.4 ± 0.1	10.1 ± 1.5
Time to Maximum Volume (hr)	36.2 ± 6.1	27.8 ± 8.8	42.6 ± 0.5	29.6 ± 13.4	25.7 ± 0	35.2 ± 8.7
Volume of CO_2 at Maximum Rate (mL)	35.9 ± 4.3	34.1 ± 0.8	32.0 ± 9.7	25.3 ± 7.2	27.2 ± 4.7	29.9 ± 3.4
Maximum CO_2 Volume (mL)	53.9 ± 4.6	50.1 ± 2.7	47.7 ± 10.2	40.4 ± 7.7	37.8 ± 3.5	52.4 ± 12.0
**Shake Flask**						
Final OD	3.6 ± 2.2	5 ± 0.6	3.1 ± 2.2	4.9 ± 0.3	4.7 ± 0.3	5.8 ± 1.7
Glucose Consumption (%)	75.1 ± 49.9	100 ± 0.0	60.6 ± 43.9	100 ± 0.0	100 ± 0.0	100 ± 0.0
Xylose Consumption (%)	8.6 ± 11.5	27.0 ± 8.0	-3.6 ± 5.1	21.9 ± 6.6	37.7 ± 4.2	23.1 ± 12.3
Glucose Consumption (g/L)	47.9 ± 32.0	48.2 ± 2.0	28.5 ± 20.7	53.6 ± 0.8	44.5 ± 0.7	55.9 ± 0.5
Xylose Consumption (g/L)	3.0 ± 3.9	9.1 ± 2.8	-1.2 ± 1.7	7.5 ± 2.3	11.5 ± 1.1	7.6 ± 4.0
Ethanol Produced (g/L)	24.1 ± 16.2	27.3 ± 2.4	15.4 ± 10.7	28.2 ± 1.3	25.6 ± 1.2	29.9 ± 2.4
Process Ethanol Yield (%)[§]	48.6 ± 32.3	65.4 ± 4.2	37.9 ± 26.2	63.2 ± 3.5	66.9 ± 4.1	65.8 ± 5.6
Metabolic Ethanol Yield (%)[‡]	72.6 ± 44.1	93.4 ± 3.2	115.6 ± 12.8	90.9 ± 7.3	89.6 ± 3.7	92.1 ± 2.5
Maximum Rate of CO_2 Production (µL/hr)	1.3 ± 0.6	1.9 ± 0.1	1.4 ± 0.6	1.6 ± 0.1	1.8 ± 0.3	1.7 ± 0.4
Time to Maximum Rate of CO_2 Production (hr)	17.8 ± 15.5	12.2 ± 2.5	42.0 ± 0.0	19.5 ± 3.6	11.3 ± 1.5	11.6 ± 1.5
Time to Maximum Volume (hr)	28.2 ± 13.9	35.7 ± 5.4	42.6 ± 0.5	35.9 ± 5.8	35.7 ± 5.5	31.6 ± 5.1
Volume of CO_2 at Maximum Rate (mL)	33.0 ± 21.5	37.3 ± 3.0	26.8 ± 15.7	33.1 ± 3.5	34.4 ± 1.4	34.2 ± 4.2
Maximum CO_2 Volume (mL)	49.2 ± 31.5	56.3 ± 0.1	29.5 ± 17.3	50.0 ± 5.9	51.9 ± 9.1	56.7 ± 12.2

[‡]The metabolic yield is the ratio of sugars (glucose and xylose) consumed during fermentation to ethanol produced assuming 0.51 g ethanol/g sugars as the theoretical maximum.
[§]The process yield is the ratio of sugars initially present in the hydrolysate (glucose and xylose) to ethanol produced assuming 0.51 g ethanol/g sugars as the theoretical maximum.

Table S3: Summary of *Zymomonas mobilis* 2032 fermentation results. Values are reported as the mean ± s.d. (n=3 or 4).

	2008 CS	2010 SG	2012 SG	2014 MG	2014 RP	2014 SO
**Roller Bottle**						
Final OD	2.7 ± 1.4	3.0 ± 0.2	2.9 ± 0.6	3.3 ± 0.5	3.8 ± 0.3	3.4 ± 0.5
Glucose Consumption (%)	78.5 ± 43.0	100 ± 0.0	100 ± 0.0	100 ± 0.0	100 ± 0.0	100 ± 0.0
Xylose Consumption (%)	51.0 ± 32.8	90.1 ± 4.8	75.4 ± 20.0	81.7 ± 10.1	86.7 ± 0.5	81.8 ± 2.3
Glucose Consumption (g/L)	47.4 ± 25.9	49.2 ± 6.2	45.8 ± 0.8	45.5 ± 2.5	46.8 ± 2.3	53.6 ± 6.6
Xylose Consumption (g/L)	14.7 ± 9.4	31.4 ± 4.4	21.8 ± 5.6	22.7 ± 4.2	27.4 ± 0.7	24.3 ± 3.3
Ethanol Produced (g/L)	26.1 ± 16.7	31.8 ± 0.5	30.0 ± 2.2	29.3 ± 3.0	33.2 ± 0.4	32.0 ± 0.8
Process Ethanol Yield (%)[§]	57.4 ± 37.0	74.9 ± 9.1	78.8 ± 6.0	78.4 ± 4.0	83.2 ± 2.5	76.3 ± 10.2
Metabolic Ethanol Yield (%)[‡]	69.5 ± 31.8	78.3 ± 10.9	87.1 ± 0.9	84.2 ± 1.9	87.9 ± 2.5	81.6 ± 11.6
Maximum Rate of CO_2 Production (µL/hr)	1.2 ± 0.6	1.8 ± 0.1	1.7 ± 0.6	1.5 ± 0.1	1.9 ± 0.1	1.8 ± 0.1
Time to Maximum Rate of CO_2 Production (hr)	16 ± 13.2	14.9 ± 4.8	16.3 ± 4.7	21.8 ± 6.6	11.9 ± 0.1	15.4 ± 3.4
Time to Maximum Volume (hr)	39.4 ± 3.9	39.6 ± 4.7	37.2 ± 9.1	38.2 ± 4.7	36.2 ± 0.5	38.6 ± 3.3
Volume of CO_2 at Maximum Rate (mL)	28.6 ± 18.9	35.9 ± 2.8	36.0 ± 14.1	38.5 ± 5.1	35.2 ± 3.2	39.2 ± 5.0
Maximum CO_2 Volume (mL)	45.3 ± 27.1	60.1 ± 2.1	55.8 ± 17.1	53.2 ± 5.9	54.3 ± 5.9	59.0 ± 6.3
**Shake Flask**						
Final OD	2.2 ± 1.1	2.8 ± 0.4	3.0 ± 0.3	2.6 ± 1.0	3.6 ± 0.5	3.3 ± 0.7
Glucose Consumption (%)	73.6 ± 45.7	100 ± 0.0	100 ± 0.0	75.5 ± 40.7	100 ± 0.0	100 ± 0.0
Xylose Consumption (%)	35.0 ± 22.3	76.8 ± 10	72.1 ± 3.5	23.4 ± 26	85.9 ± 2.2	62.5 ± 12.4
Glucose Consumption (g/L)	46.5 ± 28.5	48.2 ± 2.0	47.5 ± 0.8	40.3 ± 21.5	44.5 ± 0.7	55.9 ± 0.5
Xylose Consumption (g/L)	11.5 ± 7.1	25.8 ± 3.6	23.8 ± 1.4	7.9 ± 8.8	26.3 ± 1.1	20.6 ± 3.7
Ethanol Produced (g/L)	27.5 ± 19.3	35.7 ± 2.1	35.3 ± 0.3	24.1 ± 14.2	34.5 ± 2.1	37.4 ± 3.0
Process Ethanol Yield (%)[§]	56.2 ± 39.9	85.7 ± 2.5	86.1 ± 1.8	54.2 ± 32.1	90.0 ± 5.2	82.5 ± 6.8
Metabolic Ethanol Yield (%)[‡]	84.5 ± 20.9	94.8 ± 2.8	97.2 ± 3.1	96.7 ± 4.5	95.5 ± 4.7	95.8 ± 2.4
Maximum Rate of CO_2 Production (µL/hr)	1.1 ± 0.4	1.6 ± 0.2	1.5 ± 0.3	1.3 ± 0.4	1.9 ± 0.1	1.6 ± 0.1
Time to Maximum Rate of CO_2 Production (hr)	16.3 ± 14.0	17.9 ± 5.0	19.1 ± 5.7	37.7 ± 7.0	18.5 ± 2.5	18.8 ± 6.9
Time to Maximum Volume (hr)	42.6 ± 0.4	41.4 ± 2.1	42.3 ± 1.0	42.6 ± 0.3	41.9 ± 0.4	42.1 ± 0.3
Volume of CO_2 at Maximum Rate (mL)	30.3 ± 24.9	40.6 ± 1.3	34.5 ± 4.3	36.0 ± 10.4	41.0 ± 4.2	39.6 ± 4.1
Maximum CO_2 Volume (mL)	51.7 ± 30.7	64.7 ± 2.0	57.0 ± 9.1	43.2 ± 17.9	62.3 ± 4.2	64.2 ± 5.0

[‡]The metabolic yield is the ratio of sugars (glucose and xylose) consumed during fermentation to ethanol produced assuming 0.51 g ethanol/g sugars as the theoretical maximum.
[§]The process yield is the ratio of sugars initially present in the hydrolysate (glucose and xylose) to ethanol produced assuming 0.51 g ethanol/g sugars as the theoretical maximum.

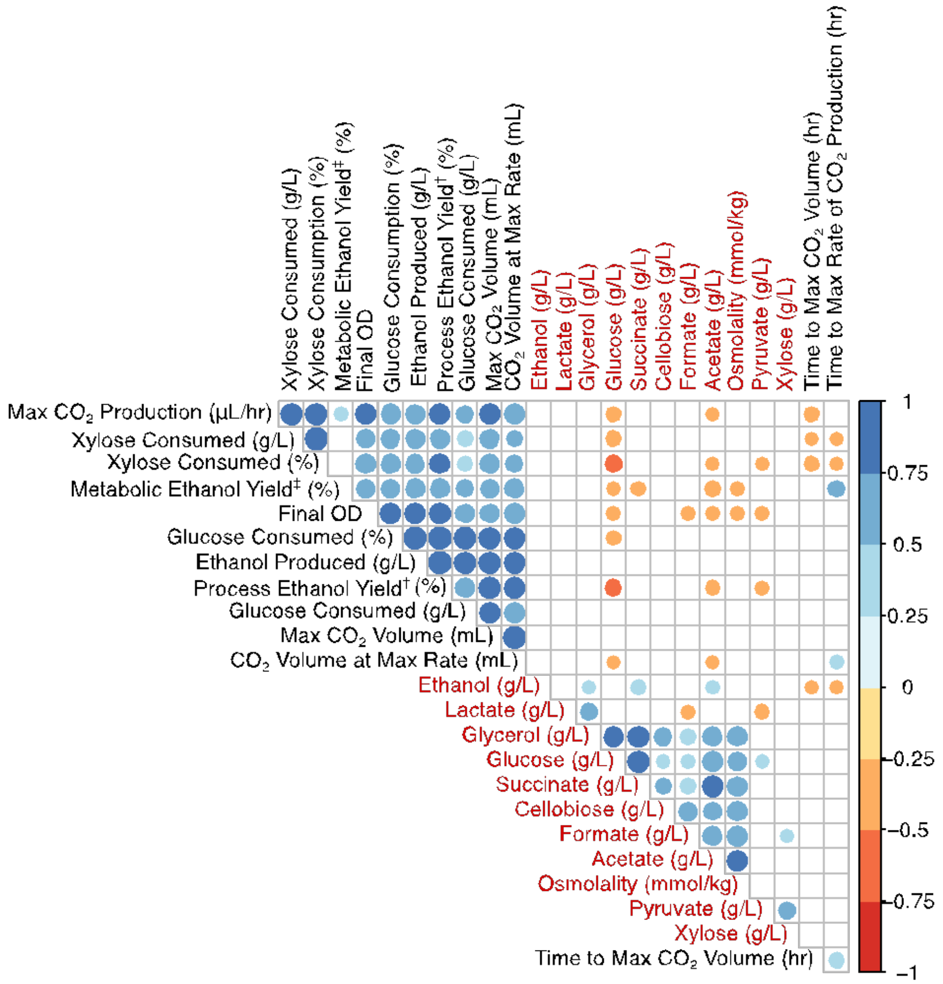

Figure S2) Clustered correlation matrix for Z. mobilis 2032 fermentation data and hydrolysate composition (red text labels). The plot was generated using the corrplot package in R with hclust (hierarchical clustering order). The size and color correspond to the direction and magnitude of the correlation. Correlations that were insignificant ($p < 0.05$) were not plotted.
